# Diagnostic value of superb microvascular imaging and color doppler for thyroid nodules: A meta-analysis

**DOI:** 10.3389/fonc.2023.1029936

**Published:** 2023-04-05

**Authors:** Haorou Luo, Lixue Yin

**Affiliations:** ^1^ Chengdu Women’s and Children’s Central Hospital, School of Medicine, University of Electronic Science and Technology of China, Chengdu, Sichuan, China; ^2^ Sichuan Academy of Medical Sciences, Sichuan Provincial People’s Hospital, School of Medicine University of Electronic Science and Technology of China (UESTC), Chengdu, Sichuan, China

**Keywords:** thyroid nodules, superb microvascular imaging, ultrasonography, meta-analysis, SMI = superb microvascular imaging

## Abstract

**Objective:**

Superb micro-vascular imaging (SMI) is a new noninvasive modality for the diagnosis of thyroid nodules. However, the performance of SMI in differentiating malignant and benign thyroid nodules has not been systematically evaluated. This meta-analysis was performed to assess the accuracy of SMI in diagnosing thyroid nodules.

**Methods:**

PubMed, Cochrane Library, Embase, Web of Science, Sinomed, Scopus were searched. We recorded the characteristics of the included studies and assessed the quality of each study using the QUADAS-2 tool. The pooled sensitivity, specificity, positive likelihood ratio (LR), negative LR, diagnostic odds ratio (DOR), and area under the curve (AUC) were calculated. We also evaluated the publication bias.

**Results:**

This meta-analysis included 10 studies with a total of 1083 thyroid nodules. The pooled the sensitivity, specificity, and positive and negative LR were 0.84, 0.86, 6.2, and 0.18, respectively. The DOR and AUC were 33 and 0.91, respectively. Heterogeneity existed between the included studies. No significant publication bias was observed.

**Conclusion:**

Compared with CDFI, Superb micro-vascular imaging (SMI) has higher diagnostic sensitivity and specificity, better diagnostic efficiency, and could be used to diagnose benign and malignant nodules in the display of blood flow distribution capabilities of thyroid nodules; at the same time, Fagan plot showed that the SMI technique had a good clinical application value, and it could supplement the deficiencies of color Doppler imaging in the diagnosis of thyroid nodules.

## Introduction

Thyroid nodules are discrete lesions in the thyroid gland that are distinct from the surrounding parenchyma (1). Recently, the incidence of thyroid nodule has been increasing year by year, making it one of the most common diseases of the thyroid system, with occult thyroid nodule accounting for 65% of the total population (2). According to the report, approximately 5% of thyroid nodules are at risk of developing thyroid cancer (3), with thyroid carcinoma becoming the most common tumour in the head and neck region (4). In clinical practice, ultrasound is the most sensitive imaging method for diagnosing thyroid nodules (5). According to research, it is necessary to evaluate the blood supply in thyroid nodules and distinguish benign from malignant nodules (6).

As a new angiography technology, superb microvascular imaging (SMI) can visualise low-speed blood flow and depict vascular flow in more detail than colour Doppler flow imaging, allowing for higher-quality microvascular flow images without the use of contrast media (7, 8). In recent years, with the widespread use of SMI technology in breast, liver and other fields, an increasing number of research teams have begun to pay attention to the diagnostic value of SMI in thyroid nodules. Despite numerous studies comparing ultrasound microangiography to conventional two-dimensional ultrasound diagnosis, there are still internal debates regarding the comparison of sensitivity, specificity and other indicators. Therefore, in order to provide a relatively objective and comprehensive evidence-based diagnostic basis for clinical thyroid management, this study collected all relevant literatures from both domestic and international sources, summarised the data of the two diagnostic methods using meta-analysis and comprehensively compared the diagnostic efficacy with pathological diagnosis as the reference standard.

## Review methods

This present study has been conducted and reported in accordance with the PRISMA statement. Institutional review board approval was not required as our study was a meta-analysis and consent was not required for this research.

## Literature search

We conducted a comprehensive search of all literatures in PubMed, Cochrane Library, Embase, Web of Science, Sinomed, Scopus and other databases until January 2021. Searches were conducted using the following MeSH heading and key words: “thyroid nodules[MeSH] OR thyroid disease OR thyroid” and”Superb Microvascular Imaging OR SMI”. In order to expand the scope of our search, we also screened the references of the retrieved articles. Language restrictions are not applied.

## Inspection methods and study selection

Routine two-dimensional ultrasonography was performed on the patients, and the thyroid nodule was observed in terms of boundary, size, internal echo, morphology, calcification, etc., and was preliminarily classified according to the TI-RADS classification system (1). Superb Microvascular Imaging (SMI) and color Doppler flow imaging (CDFI) were used to evaluate the nodules on the basis of conventional two-dimensional imaging. The blood supply and blood flow distribution pattern of the nodules were mainly evaluated. The classification standard of blood flow distribution pattern was based on Kim semi-quantitative method (9), and the blood flow signal grading was based on Adler classification method (10).

The selection of the literature by the two personnel was carried out according to the inclusion and exclusion criteria for diagnostic trials recommended by Cochrane. The process was ensured to be independent and separate. Finally, the two data were obtained, and then the data were cross-checked.

Inclusion criteria: (1) The pathological diagnosis results were considered as the “gold standard”, and the disease diagnosis was clear; (2) Subjects included patients with thyroid nodule with other diseases, whose nature was not defined before thyroid nodule examination; (3) Data sample size ≥30 cases; (4) Microangiography and conventional two-dimensional ultrasonography were in the same group; (5) The literatures of TP, FP, FN and TN can be directly extracted or indirectly calculated according to sensitivity, specificity and number of cases.

Exclusion criteria: (1) No literature on pathological diagnosis; (2) Literature that was directly included in the study with cases after diagnosis; (3) Reviews, conferences, case reports; (4) Incomplete data, TP, FP, FN and TN cannot be directly extracted or indirectly calculated according to sensitivity, specificity and number of cases; (5) Duplicate literature searched in different databases.

## Data extraction and quality assessment

The data for qualified full-text articles were independently extracted by two researchers (Haorou, Luo and Lixue, Yin), and different comments were solved by discussion and consensus. The recorded data are as follows: First author, language, year, mean age of patients, number of malignant lesions, “gold standard” diagnosis, type of study. The diagnostic trials were analyzed according to the Cochrane recommended diagnostic accuracy study quality assessment tool (QUADAS-2) (11), and then the risk of bias and clinical applicability were evaluated summatively.

## Statistical analyses

The heterogeneity of the included literatures were evaluated using the inconsistency index (I^2^) and Cochrane Q-tests(x^2^). If I^2^ > is 50% or P < 0.10, high heterogeneity exists among the results. I^2^ < 25% indicates that there is little heterogeneity among the results. 25%≤I^2^ ≤ 50%, indicating moderate heterogeneity of the results. At the same time, the Bivariate boxplot can be used to make rough judgment, and then guide the sensitivity analysis. If the heterogeneity result is large, try to trace the source of heterogeneity by means of meta-regression. The positive likelihood ratio, negative likelihood ratio, sensitivity, specificity and diagnostic odds ratio of the two methods were analyzed and compared, and then the SROC curve was drawn. Sensitivity analysis and meta-regression subgroup analysis were performed. Deek funnel plot was drawn to evaluate whether there was publication bias, and the funnel plot was observed to be symmetrical, combined with P value to make a comprehensive evaluation. Fagan plot can show the correlation between prior probability, likelihood ratio and post-test probability. The greater the difference between prior and post-test probability, the more important the diagnostic test is, and it can provide a reference for clinical application evaluation. All analyses were completed using StataV 15.0.

## Results

### Literature search and selection

**Figure 1:**
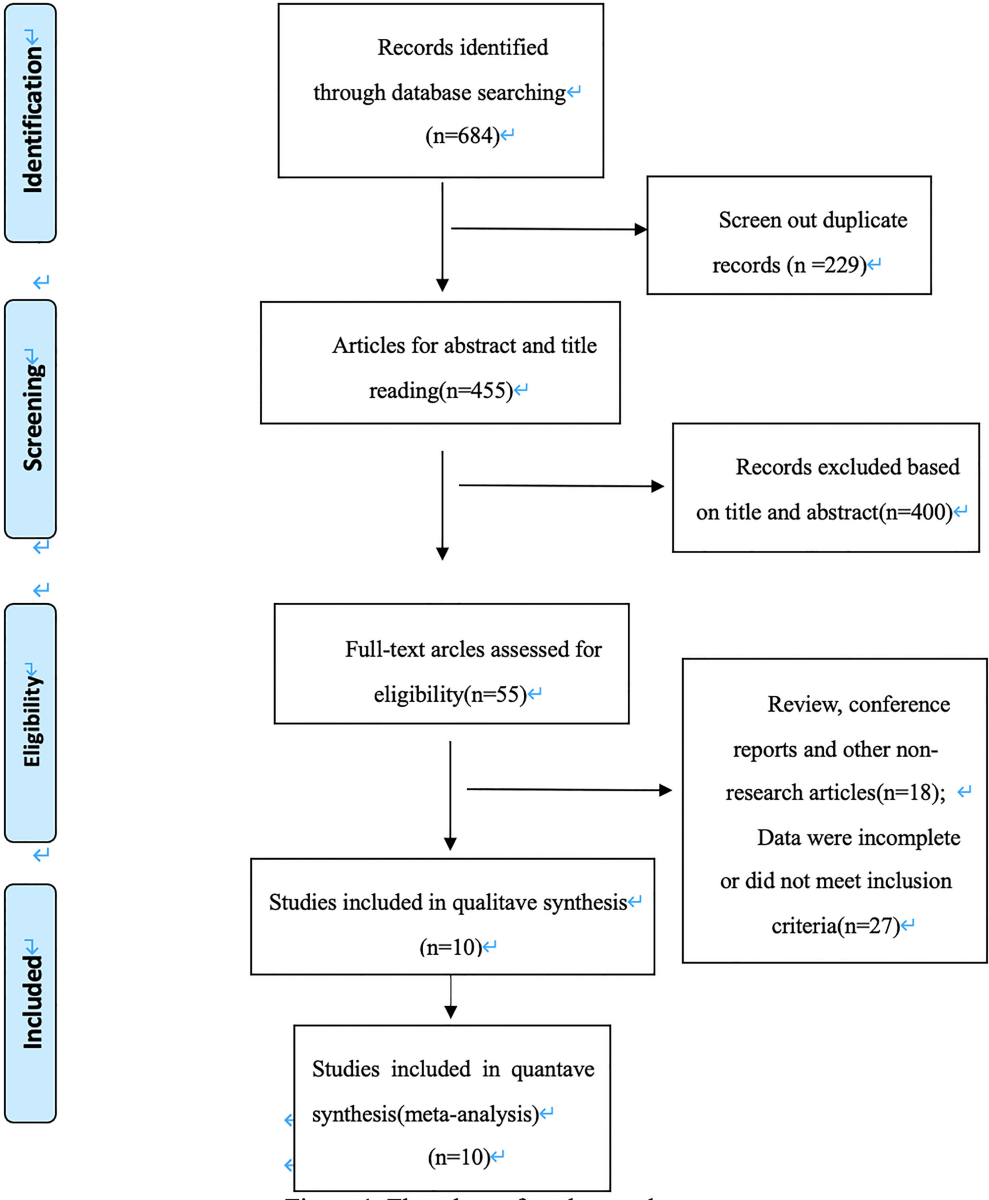
Flowchart of study search.

As shown in **Figure 1**, the initial search yielded 684 records. 229 records were excluded after removing duplicates. and 400 were excluded after screening the titles and abstracts. Depending on the inclusion and exclusion criteria, 45 records were deleted, ten studies ultimately are included in our meta-analysis (12–21).

### Study characteristics

The included 10 records included patients who were examined by both ultrasound microvascular imaging and color Doppler blood flow imaging, with a total of 1083 lesions, of which 518 were malignant lesions. Details of the eligible studies are shown in **Table 1**. All of them were diagnostic studies published from 2016 through 2020, 5 of which were recorded in Chinese and 5 in English; Nine records were prospective studies, and only one record was retrospective studies; The gold standard for all the selected studies was pathological tissue study, of which 3 records were surgical pathology plus fine needle aspiration pathology. The average age of all included patients was under 55. The number of lesions varied from 45 to 254, and the number of patients varied from 34 to 241.

**Table 1:** characteristics of the included studies.

Author	Year	Country	Language	average age	Total Lesions	Malignant Lesions	Male/Female	Gold Standard	Study design
**Tian J**	2017	China	Chinese	47.3 ± 10.8	45	25	8/26	surgical Pathology	Prospective cohort
**Ouyang**	2017	China	Chinese	39.2 ± 11.5	76	41	16/41	surgical Pathology	Prospective cohort
**Diao XH**	2016	China	Chinese	44.8 ± 17.6	68	27	11/36	surgical Pathology	Prospective cohort
**Yang HX**	2017	China	Chinese	47.1 ± 8.2	83	28	18/42	surgical Pathology	Prospective cohort
**Wang H**	2020	China	Chinese	38.9 ± 12.2	120	72	13/91	surgical Pathology	Retrospective cohort
**Li YH**	2017	China	English	39.0 ± 16.5	254	73	53/188	surgical Pathology+FNA	Prospective cohort
**Kong J**	2017	China	English	42	113	79	48/44	surgical Pathology	Prospective cohort
**Zhu YC**	2018	China	English	49.6 ± 13.2	76	29	35/41	surgical Pathology+FNA	Prospective cohort
**Hye Shin**	2017	South Korea	English	51.6 ± 11.2	52	26	9/48	surgical Pathology+FNA	Prospective cohort
**Pei SF**	2019	China	English	--	196	118	58/112	surgical Pathology	Retrospective cohort

### Quality assessment

The quality assessment of the included studies were evaluated as follows (**Table 2**). All included studies were in compliance with the index test standards, with pathological evaluation as the reference standard.

**Table 2:** Quality assessment of the included studies using QUADAS-2 tool.

References	Risk of bias				Applicability concerns		
	Patient selection	Index test	Reference standard	Flow and timing	Patient selection	Index test	Reference standard
**Tian J**	U	L	L	L	L	L	L
**Ouyang**	L	L	U	L	L	L	L
**Diao XH**	U	L	L	L	U	L	L
**Yang HX**	L	L	U	L	L	L	L
**Wang H**	L	H	H	L	U	L	L
**Li YH**	L	L	L	H	L	L	L
**Kong J**	L	L	L	L	L	L	L
**Zhu YC**	L	L	L	H	L	L	L
**Hye Shin**	L	L	L	L	L	L	L
**Pei SF**	L	L	L	L	L	L	L

*. L, low risk of bias; H, high risk of bias; U, unclear risk of bias.

### Diagnostic performance

**Figure 2:**
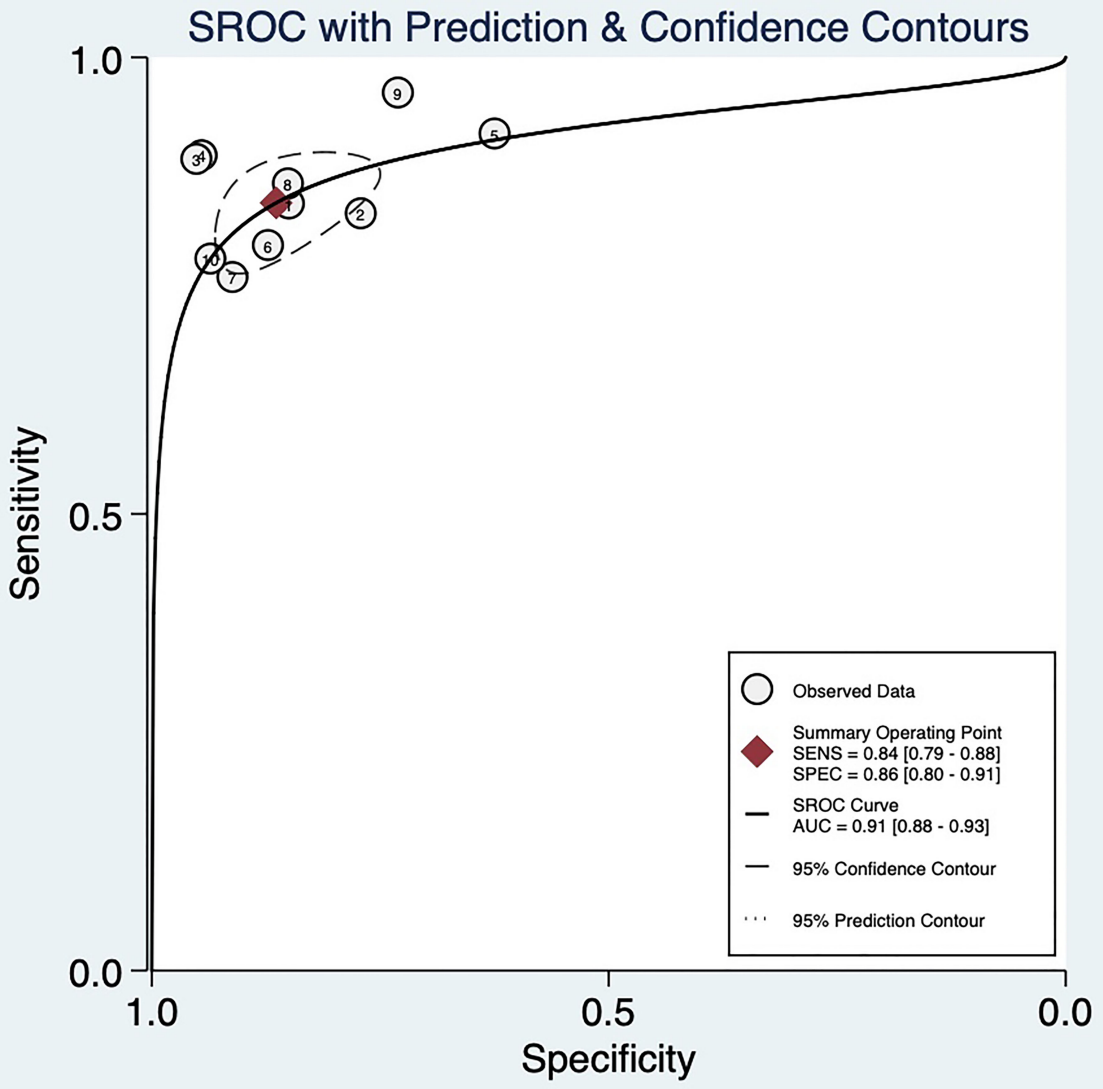
SROC curve and 95% confidence region.

**Figure 3:**
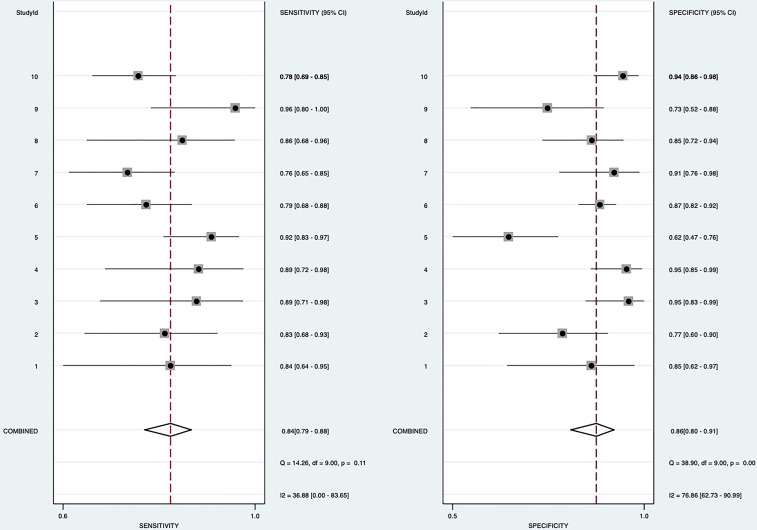
Forest plot for sensitivity and specificity.

**Figure 4:**
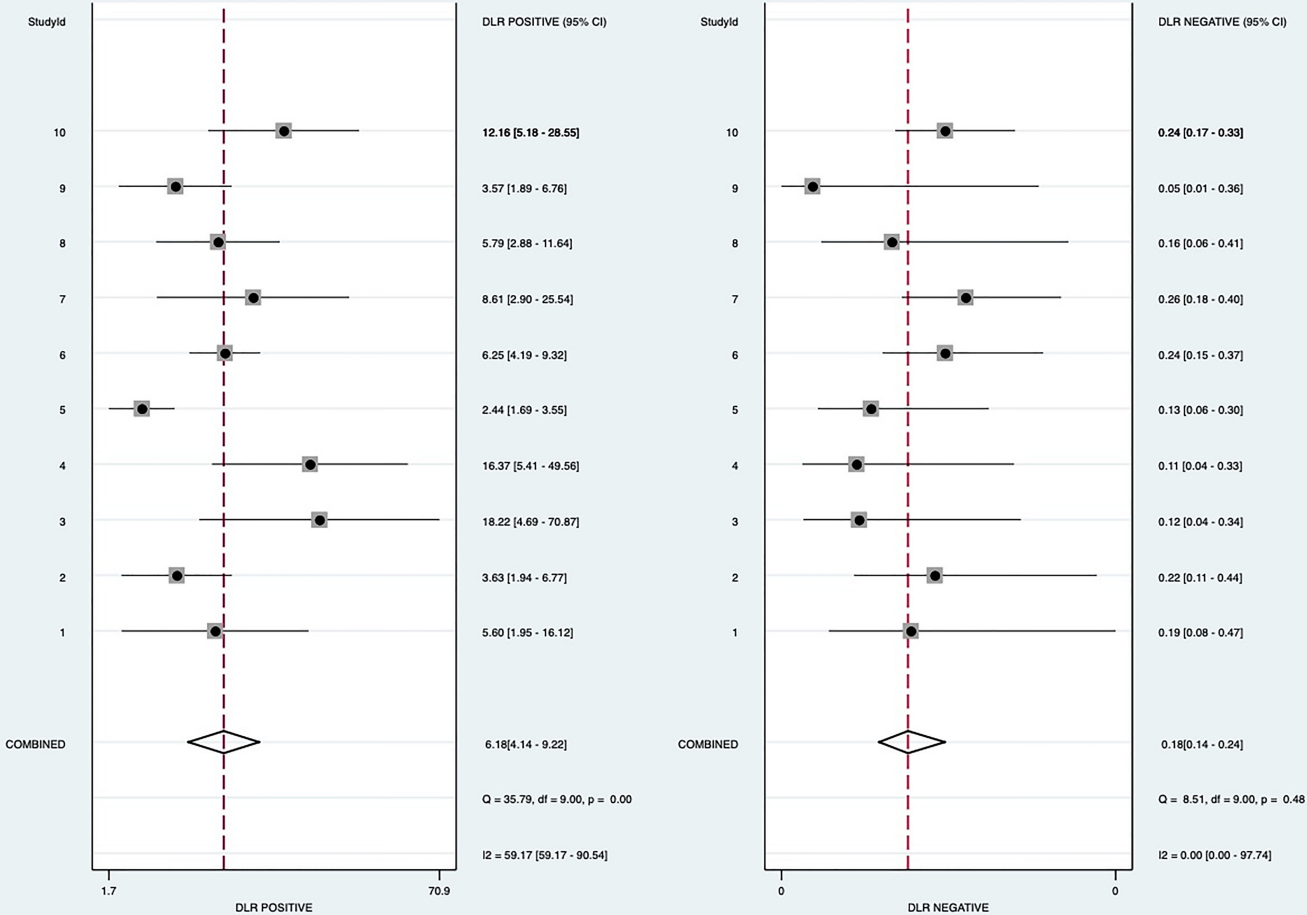
Forest plot for Positive likelihood ratio and negative likelihood ratio.

**Figure 5:**
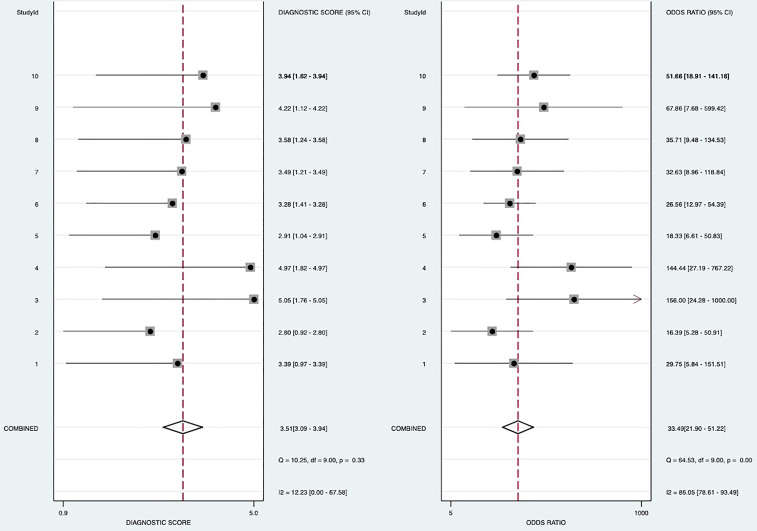
Forest plot for Odds ratio.

Random-effects model was used to generate the pooled result. The pooled sensitivity, specificity, positive LR, and negative LR of SMI in differentiating malignant and benign thyroid nodules were 0.84 (95% confidence interval [CI], 0.79-0.88), 0.86 (95% CI, 0.80-0.91), 6.2 (95% CI, 4.1-9.2), and 0.18 (95% CI, 0.14-0.24), respectively. The area under the curve (AUC) was 0.91 (**Figure 2**). The forest plots of sensitivity, specificity, positive LR, negative LR, and DOR of the included studies were described in **Figures 3**–**5**. The detailed comparison of the combined effect size SMI and color Doppler was shown in **Table 3**.

**Table 3:** Comparison of combined effects.

	sensitivity	specificity	PLR	NLR	DOR	AUROC
**SMI**	0.84(0.79-0.88)	0.86(0.80-0.91)	6.2(4.1-9.2)	0.18(0.14-0.24)	33(22-51)	0.91(0.88-0.93)
**CDFI**	0.64(0.56-0.71)	0.78(0.72-0.83)	2.8(2.3-3.5)	0.47(0.39-0.56)	6(4-9)	0.77(0.74-0.81)

### Heterogeneity analyses

When all 10 included studies were analyzed, heterogeneity was found both in sensitivity (heterogeneity, Q=14.26, p =0.11; I^2 =^ 36.88) and specificity (heterogeneity, Q =38.90, p < 0.0001; I^2 =^ 76.86%). To trace the sources of heterogeneity, we primarily analyzed the threshold effect. But there was no shoulder-arm sign shown on the sROC space (**Figure 2**), and the Spearman correlation coefficient was 0.349 (P=0.324), indicating that all records had no significant threshold effect, and the effect size could be combined. It also indicated that there were other factors leading to heterogeneity in the inclusion analysis. The results of the subgroup analyses showed in **Table 4**. For the bivariate box chart, the documents outside the central area were deleted and then meta-analysed. It was found that the combined effect of various indicators had little change, indicating that the included documents were stable

**Table 4:** Meta regression analysis results.

Subgroup	Number of studies	Pooled sensitivity	P1	Pooled specificity	P2	I^2^ value,%
**Prospective**	8	0.84(0.79-0.89)	0.00	0.87(0.81-0.93)	0.46	0
**Retrospective**	2	0.85(0.78-0.93)	–	0.82(0.68-0.97)	–	–
**Lesions≥100**	4	0.82(0.76-0.88)	0.00	0.86(0.78-0.95)	0.05	30
**Lesions ≤ 100**	6	0.88(0.82-0.93)	–	0.87(0.79-0.94)	–	–
**English**	5	0.81(0.76-0.87)	0.00	0.88(0.80-0.95)	0.12	6
**Chinese**	5	0.87(0.82-0.93)	–	0.85(0.76-0.94)	–	77
**Surgical pathology**	7	0.83(0.78-0.89)	0.00	0.88(0.82-0.94)	0.30	0
**Surgical pathology+FNA**	3	0.85(0.78-0.93)	–	0.83(0.71-0.95)	–	–
**Chinese**	9	0.83(0.79-0.87)	0.14	0.87(0.82-0.93)	0.53	48
**South Koreans**	1	0.96(0.89-1.00)	–	0.74(0.45-1.00)	–	–

### Publication bias

**Figure 6:**
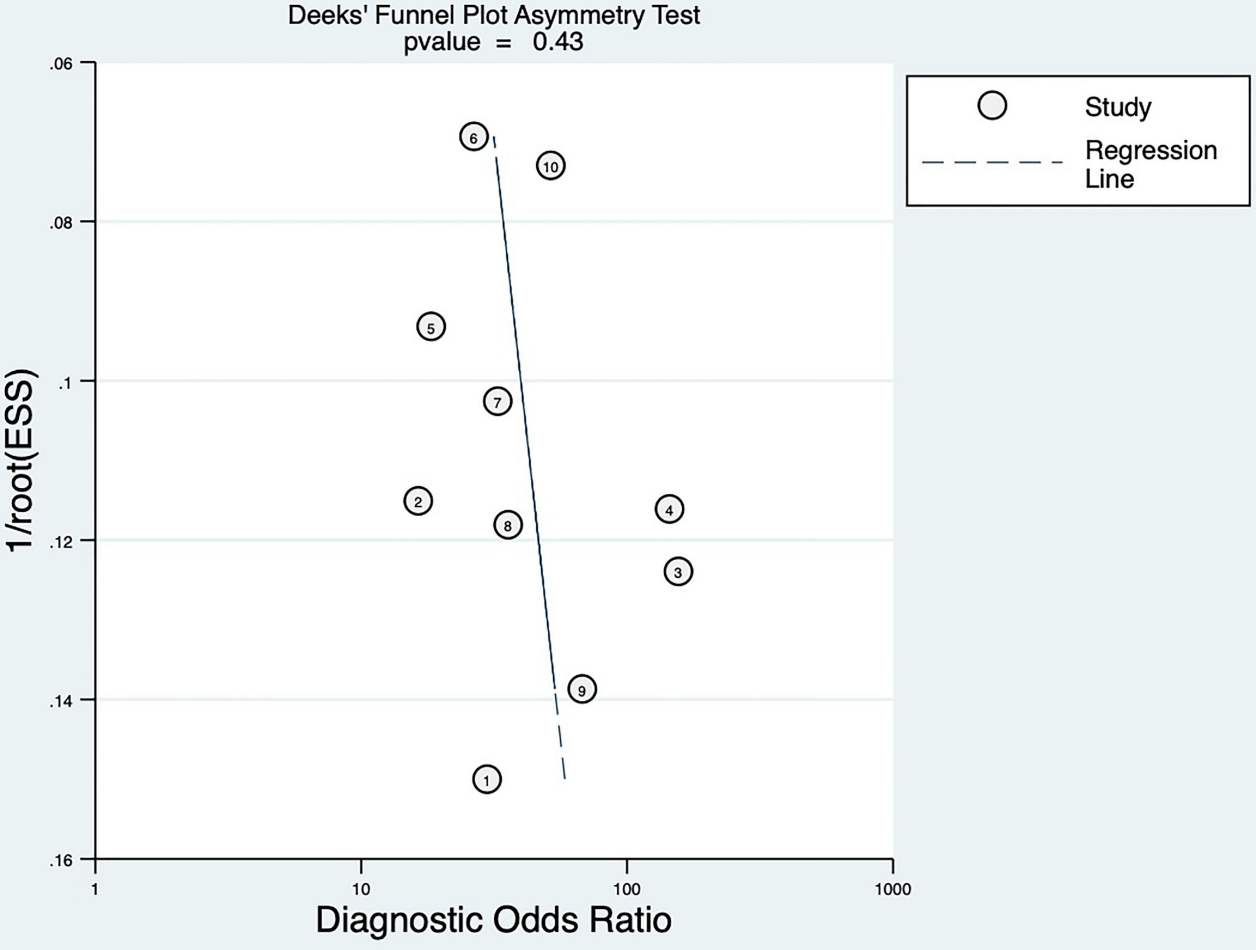
Deek’s funnel plot of publication bias.

By Deeks’ funnel plot asymmetry test, we found there was no significant publication bias among the eligible studies (P = 0.43). The Deeks’ funnel plot was shown in **Figure 6**.

### Clinical application evaluation

**Figure 7:**
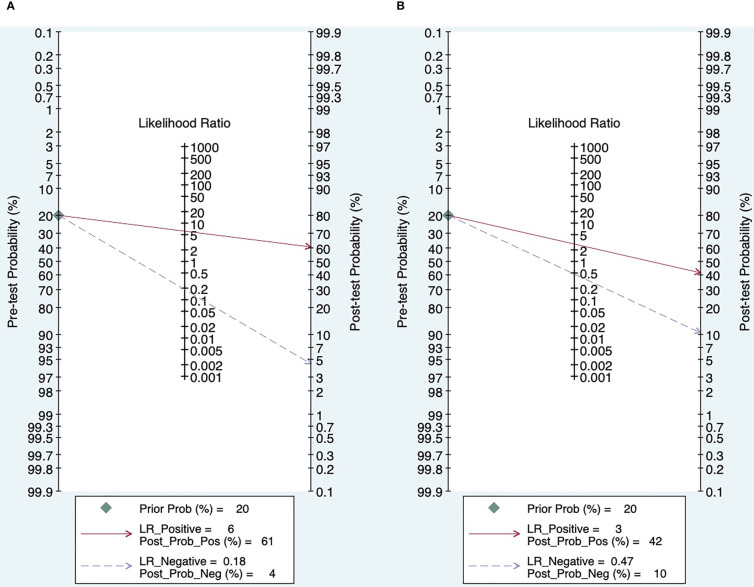
Fagan’s plot (**A**. SMI; **B**. CDFI).

According to Fagan’s plot, the post-test probability of SMI was significantly higher than that of CDFI (**Figure 7**).

## Discussion

Thyroid nodules have been detected in up to 65% of the total population, indicating that thyroid nodules have become a very common disease (22). The majority of nodules detected by us are benign and can be safely managed through monitoring procedures. The purpose of initial screening and long-term follow-up of thyroid nodules is to better manage the most critical thyroid malignancies (2).

Fine needle aspiration biopsy has always been used to determine the nature of nodules related to clinical malignancies, and its status as the gold standard is unshakable (23). However, routine physical examination requires a non-invasive diagnostic method to assess the characteristics of blood flow distribution and understand the characteristics of blood supply in order to guide puncture (24).

Thyroid ultrasound is considered to be the best imaging method for assessing thyroid nodules or diffuse disease (25), Over the past 25 years, significant advances have been made in the evaluation of the thyroid by ultrasound and in the identification of suspicious and non-suspicious thyroid nodules (26). However, in general, the current conventional color Doppler blood flow imaging still has the disadvantages of insufficient accuracy, a large number of misdiagnosis, and missed diagnosis in the identification of the nature of thyroid nodules. And there are technical limitations to detecting small blood vessels and low blood flow.

Angiogenesis and the growth of irregular vascular structures have been established as the salient features of malignancy. Several previously published studies had shown that peri-nodular blood flow was more associated with benign thyroid nodules, while intra - nodular blood flow tended to be present in malignancies. Therefore, changes in vascular characteristics may provide more valuable diagnostic clues (9, 27).

In recent years, the rapid development of Superb micro-vascular imaging aimed at the deficiency of two-dimensional gray scale ultrasound and color Doppler blood flow imaging, and provided information about low speed blood flow, detection of small vessels (8). It reveals more microvascular branches, and shows the blood flow distribution in the nodules and adjacent thyroid parenchyma in more detail, which has become a more effective detection method (28). Although Superb micro-vascular imaging could be used to distinguish malignant and benign thyroid nodules, the value of its differential diagnosis is still inconclusive, and there is currently no Meta-analysis on the diagnostic performance of SMI technology. Therefore, the method of systematic review in this study was adopted to compare the value of SMI and CDFI in differentiating benign and malignant thyroid nodules. To provide an exact evidence-based basis for the differential diagnosis of benign and malignant thyroid nodules by SMI technology, so as to better guide the clinical diagnosis and treatment.

The pooled results showed that SMI could diagnose thyroid nodules, and the pooled sensitivity, specificity, positive LR and negative LR values were 0.84, 0.86, 6.2, and 0.18, respectively. These results indicated SMI had high sensitivity and specificity in diagnosing thyroid nodules. The AUC was 0.91, and the DOR was 33, which were the overall evaluation indicators of diagnostic tests, which proved the high accuracy of SMI in the differentiation of thyroid malignant nodules and benign nodules.

The heterogeneity test of our study showed moderate heterogeneity. We discovered that there was no significant threshold effect among the included records. Therefore, subgroup analyses were performed to find the source of heterogeneity. The results of the subgroup analyses showed that the heterogeneity of the combined estimates was reduced after excluding the Chinese study. The reassessed pooled results still suggested that SMI was more accurate in the diagnosis of thyroid nodules (AUC = 0.91, DOR = 32). In the 10 literatures included in this paper, although the “gold standard” was pathological examination, some were surgical biopsy histological pathology and some were cell puncture pathological results, which may also be one of the sources of heterogeneity.

Limitations of our meta-analysis: (1). Only 10 records were included in our meta-analysis. A small number of studies might reduce the effectiveness of the tests of heterogeneity and publication bias. (2). Different diagnostic criteria were used in the included studies, and neither of the two diagnostic methods was quantified and there was no unified diagnostic standard.

In conclusion, Superb micro-vascular imaging (SMI) is more sensitive, specific and effective than color doppler flow imaging in differentiating malignant and benign thyroid nodules. At the same time, SMI has good clinical value and can supplement the deficiency of CDFI in the diagnosis of thyroid nodule. SMI technology could truly reflect the blood flow distribution without the presence of blood flow signal spillover, and could be very sensitive to detect low blood flow and small blood vessels. Meanwhile, the examination cost is low, and compared with other imaging examinations, SMI technology has the advantages of non-trauma and non-radiation.

At present, as an emerging diagnostic technology, SMI still needs to develop a more detailed classification evaluation to quantify the form of blood flow distribution. At the same time, a unified diagnostic standard is also needed to guide clinical diagnosis, treatment and prognosis, To better tap the application potential of SMI diagnosis, future large-scale, controlled, multicenter studies are recommended.

## Data availability statement

The raw data supporting the conclusions of this article will be made available by the authors, without undue reservation.

## Author contributions

HL: conception and design, acquisition of data, analysis and interpretation of data, drafting and revising the article; final approval of the version to be published. LY: the corresponding author of this paper, acquisition of data, analysis and interpretation of data. revise the article. All authors contributed to the article and approved the submitted version.
